# Early infections by myxoma virus of young rabbits (*Oryctolagus cuniculus*) protected by maternal antibodies activate their immune system and enhance herd immunity in wild populations

**DOI:** 10.1186/1297-9716-45-26

**Published:** 2014-03-04

**Authors:** Stéphane Marchandeau, Dominique Pontier, Jean-Sébastien Guitton, Jérôme Letty, David Fouchet, Jacky Aubineau, Francis Berger, Yves Léonard, Alain Roobrouck, Jacqueline Gelfi, Brigitte Peralta, Stéphane Bertagnoli

**Affiliations:** 1 Office National de la Chasse et de la Faune Sauvage, Direction des études et de la recherche, 44323 Nantes, France; 2 Laboratoire de Biométrie et Biologie Evolutive UMR5558-CNRS, Université de Lyon, Université Claude Bernard Lyon 1, 69622 Villeurbanne, France; 3 LabEx ECOFECT - Eco-evolutionary dynamics of infectious diseases, Université de Lyon, 69000 Lyon, France; 4 Office National de la Chasse et de la Faune Sauvage, Direction des études et de la recherche, 34990 Juvignac, France; 5 INRA, UMR 1225 Interactions Hôtes-Agents Pathogènes, 31076 Toulouse, France; 6 Université de Toulouse, INP-ENVT, 31076 Toulouse, France

## Abstract

The role of maternal antibodies is to protect newborns against acute early infection by pathogens. This can be achieved either by preventing any infection or by allowing attenuated infections associated with activation of the immune system, the two strategies being based on different cost/benefit ratios. We carried out an epidemiological survey of myxomatosis, which is a highly lethal infectious disease, in two distant wild populations of rabbits to describe the epidemiological pattern of the disease. Detection of specific IgM and IgG enabled us to describe the pattern of immunity. We show that maternal immunity attenuates early infection of juveniles and enables activation of their immune system. This mechanism associated with steady circulation of the myxoma virus in both populations, which induces frequent reinfections of immune rabbits, leads to the maintenance of high immunity levels within populations. Thus, myxomatosis has a low impact, with most infections being asymptomatic. This work shows that infection of young rabbits protected by maternal antibodies induces attenuated disease and activates their immune system. This may play a major role in reducing the impact of a highly lethal disease when ecological conditions enable permanent circulation of the pathogen.

## Introduction

A same infectious agent may induce in vertebrates a large range of clinical signs from asymptomatic to a severe disease that may even lead to death. A first factor usually involved in the severity of a disease is the immunological status of the host. Following a primo-infection, the duration of acquired immunity is highly variable [1,2]. However, reinfections occurring before the acquired immunity has waned may lead to attenuated diseases if residual immunity is sufficient and reactivate the immune system, thereby extending the acquired immunity. Therefore, in situations where a pathogen circulates efficiently within the host population, individuals are steadily reinfected and maintain a high level of immunity. In these cases, individuals may develop only one event of severe disease at their first exposure to the pathogen, which is likely to occur early in life at a moment that is particularly critical because the newborn lack a fully efficient immune system. To limit the consequences of an early first infection, several mechanisms can attenuate its effect, in particular the transfer of maternal antibodies to the newborn [3].

Despite abundant literature on the evolution of host defense mechanisms against infectious agents (Review in [4]), the effect of the transfer of maternal immunity has attracted increasing attention only recently [5-11]. Several studies have demonstrated that maternal antibodies block the infection in the case of an early exposure to the parasite and inhibit the offspring immune response [12]. This blocking effect is responsible for unsuccessful vaccination in newborns [13-15]. In this case maternal immunity postpones infection and may change the disease dynamics [16]. However, other studies have shown that, in cases of early infection, maternal antibodies can only reduce the severity of the disease, thereby enabling the host immune system to develop acquired immunity without the cost of a severe disease [17-20], as initially proposed by Zinkernagel [21,22]. Maternal immunity may also have long-term effects: even in the absence of early infection, individuals born with maternal antibodies may display an enhanced immune response when infection occurs after maternal immunity has waned [23-25]. In addition, maternal immunity may even have positive trans-generational effects on offspring of females having received maternal antibodies from their own mother [25]. Most of these studies on maternal immunity have focused on the individual level but little is known about the potential effect of maternal immunity on disease dynamics and impact at the population level.

The European rabbit (*Oryctolagus cuniculus*) and myxomatosis system has been widely studied and is considered to be a classical example of coevolution [26,27]. Myxomatosis is a viral disease due to a Leporipoxvirus, which is responsible for a lethal disease in European rabbits and was introduced in several countries in the 1950’s to fight against wild rabbit pullulations: in 1950 in Australia after several unsuccessful attempts [28] and in 1952 in France [29] from where it spread throughout south-western Europe. The initial impact of the disease in France was strong and mortality rates were estimated at 90-95% [28,30]. Then, populations have steadily recovered as a result of a coevolution between the host and its pathogen [26,27,31-33]. Today, the impact of myxomatosis varies a lot, with populations where mortalities due to the disease are high and others with permanent and strong immunity levels where the disease has a very low impact, characterizing epizootic and enzootic patterns of the disease, respectively [34,35]. Changes in the virulence of myxoma virus (MYXV) were observed shortly after the introductions of the virus [36,37], with multiples genes being involved in changes in virulence [38]. The development of rabbit genetic resistance to the disease has been suggested in Europe [39,40] but it is controversial and anyway, of marginal effect [41-43]. In addition, development of immunity in the populations, which may be part of the coevolution process [44], is the third factor that has led to a decrease of the impact of the disease [26]. However, the characteristics of immune response in wild rabbits remain poorly documented. Its length and intensity depend on rabbits, likely under genetic control [45], and viral strains [46]. In laboratory conditions, some rabbits show detectable residual antibodies one year after inoculation of the virus [46]. In wild populations, some studies have shown that rabbits may be seropositive over longer periods, up to 19–24 months, but these long immunities could be due to undetected natural reinfections [32,47]. Maternal antibodies remain detectable up to 6 to 9 weeks after birth [47].

The persistence of high immunity levels in the populations, which is the result of an endemic pattern of the disease, has been shown in large populations [34,35,48]. In such populations, rabbits with symptoms of myxomatosis are observed throughout the year, with most of them showing mild clinical signs. These data suggest that most rabbits are partly protected at the time of their first exposure to the virus, otherwise some cases of severe disease should be observed. Modelling the mechanism described by Zinkernagel [21,22] explains these observed patterns of the disease. The mean severity of the disease depends on the transmission rate of the virus: high transmission rates lead to early infections of juveniles, steady reinfections, and maintenance of high immunity levels in the populations throughout the year [9,11]. In contrast, low transmission rates do not enable the maintenance of high immunity levels and lead to the epidemic pattern of the disease with a first infection occurring when maternal immunity has waned.

The aim of the present study was to highlight the Zinkernagel’s mechanism under natural conditions in the rabbit/myxomatosis system, which is a good model since juveniles are known to be highly susceptible to the disease and show severe disease even when infected by attenuated MYXV strains [49,50]. To this end we caught rabbits over three years in two wild populations to analyse the pattern and the role of immunity, and especially of maternal immunity, on the epidemiology and impact of myxomatosis. For this purpose, we detected specific anti- MYXV IgM and IgG, which allowed us to characterize MYXV circulation in both populations and to describe its effect on immunity at both individual and population levels, IgM being an indicator of recent infection and IgG persisting over several months after infection [47]. In agreement with Zinkernagel [21,22], we predict that if maternal immunity protects against severe myxomatosis while permitting to the juveniles to activate their own immune system, we should observe asymptomatic or mild myxomatosis in juveniles less than two months old before maternal immunity has waned. Alternatively, if maternal immunity has a blocking effect, then the first infection should lead rabbits to develop severe myxomatosis.

## Materials and methods

### Rabbit populations studied

The study was carried out on two study areas. Aubas (1°11′W, 45°4′N, Department of Dordogne) is situated in south-western France. The landscape is a cultivated open land constituted mainly of maize and walnut fields. Mean annual rainfall is 930 mm and mean annual temperature is 11.5 °C. Density is around 5 rabbits/ha in this population. Saint Benoist (1°53′ E, 48°42′ N, Department of Yvelines) is located in central France. The landscape is a cultivated open land with coppices. Farming practices, crops and field margin strips are managed for game. Mean annual rainfall is 630 mm and mean annual temperature is 10.1 °C. The density is lower (1–2 rabbits/ha) in Saint Benoist than in Aubas.

We trapped rabbits over three years to record data on myxomatosis events and on immunity (2002–2005 in Aubas, 2001–2004 in Saint Benoist). Trapping sessions were organized every five weeks and lasted two weeks each. Rabbits were caught in wire cage traps and individually marked. At each capture, each rabbit was weighed and a blood sample was taken on a strip of blotting paper [51,52]. Clinical signs of myxomatosis were also recorded. If any, they were distributed into 3 classes: severe (tumours ≥ 5 mm, presence of secondary lesions), mild (tumours < 5 mm, no secondary lesion), recovered (tumours in healing or healed). Rabbits from Aubas were recorded as Axx and rabbits from Saint Benoist as SBxx, where xx is the rabbit number.

### Age determination in juveniles

Rabbits were considered as juveniles until the beginning of the next breeding season, i.e. until the end of the calendar year of their birth. Afterwards individuals were considered as adults. To study maternal immunity, we considered maternal antibodies to be detectable up to 8 weeks. Indeed, it has been shown that they persist up to 6 to 9 weeks, depending on the individual and on the antibody titre of the mother [47]. We used the body weight to determine the age of the juveniles [53]. The mean weight of an 8 weeks old rabbit is about 430 g in females and 470 in males.

### Serological tests

The serological responses against MYXV were evaluated using enzyme-linked immunosorbent assays (ELISA), as previously described [54]. Briefly, two 6 mm-diameter discs of blotting paper impregnated with blood were rehydrated in 100 μL of phosphate-buffered saline (PBS) in a 96-well microplate and stored at 4 °C overnight. The eluate was then used directly in serological tests as equivalent to a 1/20 dilution of fresh serum. ELISA plate wells (Falcon Probind, Becton Dickinson, Le Pont de Claix, France) were coated with 1 μg of semi purified MYXV. The specific MYXV antibody binding was visualized by incubating the plate wells with alkaline phosphatase antibody conjugates (Goat anti rabbit IgM or IgG, 1:4000, Sigma, Saint Quentin Fallavier, France) at 37 °C during 60 mn. The absorbance of each sample was measured at a wavelength of 405 nm using a spectrophotometer 15 mn after addition of substrate solution (4-nitrophenyl phosphate disodium salt in 10% diethanolamine, pH 9.8) (Sigma). The eluate sample titre was expressed as the inverse of the highest dilution for which the optical density was greater than thrice the optical density of the negative serum standard. Titres ≥ 50 were considered as positive. The complete data set is given in Additional file 1.

## Results

### Early infections and immunity in juveniles

**Figure 1:**
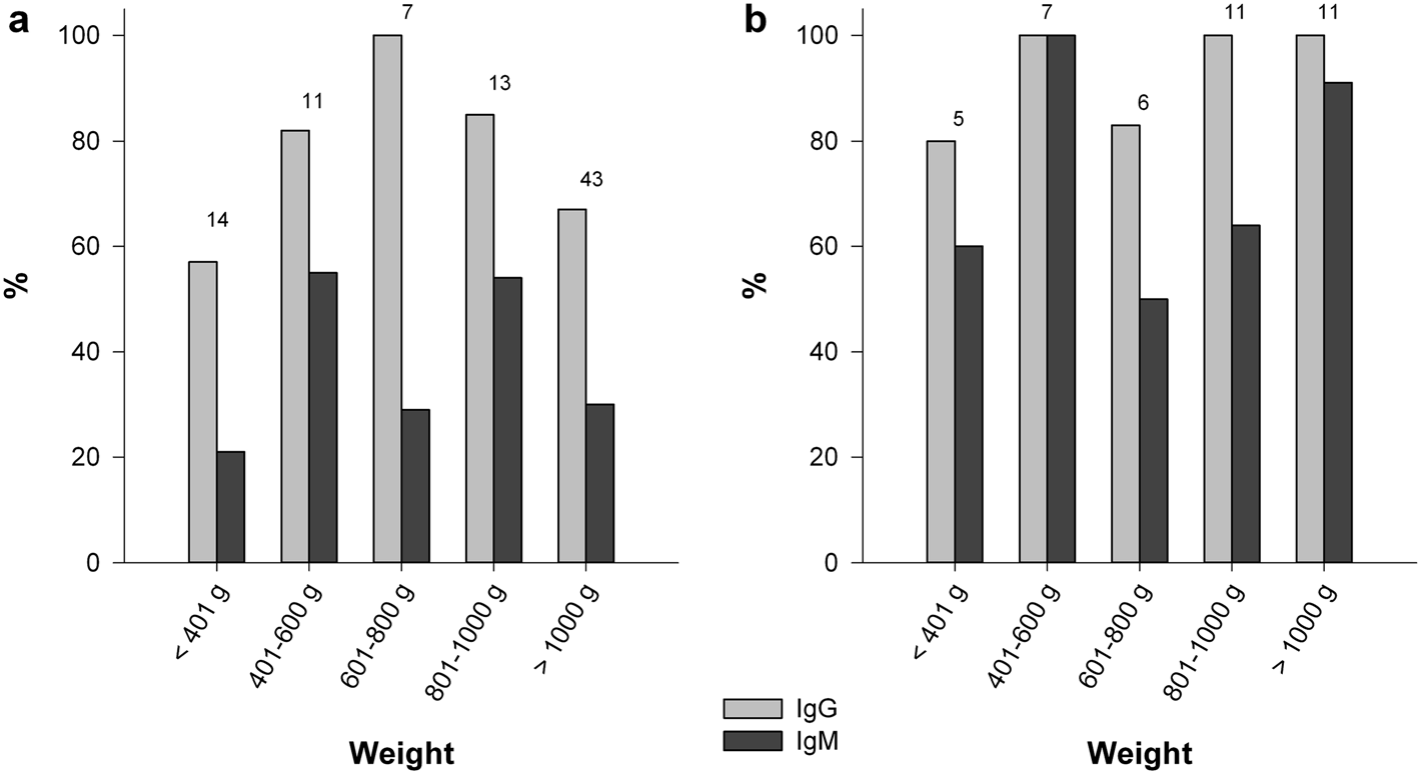
**Proportion of juveniles with IgG and IgM by weight.** Aubas: **a**. St Benoist: **b**. Sample sizes are indicated above the bars. For rabbits caught several times in the same weight class the data from the first capture was taken into account.

Most juveniles less than 8 weeks old carried IgG in both populations (57% in Aubas, 80% in Saint Benoist) (Figure 1). Among rabbits less than 8 weeks old with only IgG, none showed signs of disease or recovery (Table 1). Among those carrying both IgG and IgM (21% in Aubas, 60% in Saint Benoist) (Figure 1), which indicates a recent infection, only one showed clinical signs of myxomatosis and it developed a mild disease (Table 1).

**Table 1:** Anti MYXV-antibodies and early infection in juveniles weighing less than 600 g

**Rabbit**	**Sex**	**Date**	**Weight (g)**	**Myxomatosis**	**IgM**	**IgG**
**SB89**	F	25/06/2003	260	None	-	+
**A34**	M	23/05/2002	320	None	-	+
**A58**	F	24/09/2002	350	None	-	+
**A149**	F	21/11/2003	380	None	-	+
**A143**	M	16/10/2003	400	None	-	+
**A147**	F	18/11/2003	400	None	-	+
**A148**	M	20/11/2003	400	None	-	+
**A169**	M	17/03/2004	420	None	-	+
A144	M	17/10/2003	480	None	-	+
A185	M	11/05/2004	540	None	-	+
**A100**	F	19/03/2003	230	None	+	+
**A47**	M	23/07/2002	400	Mild	+	+
**A48**	M	23/07/2002	400	None	+	+
**SB68**	F	20/09/2002	400	None	+	+
**SB88**	F	25/06/2003	410	None	+	+
SB65	F	15/08/2002	490	None	+	+
A108	M	11/04/2003	500	None	+	+
A188	F	11/05/2004	500	None	+	+
SB48	M	23/07/2002	510	None	+	+
A164	F	15/03/2004	520	None	+	+
A184	F	11/05/2004	520	None	+	+
SB91	F	11/07/2003	550	None	+	+

In bold rabbits less than 8 weeks old, i.e. for which IgG likely have a maternal origin component.

### Reinfections and reactivation of the immune system

Several rabbits showed evidence of reinfection in both populations (Table 2). For example, rabbit A132 (adult) had only IgG on 23 September 2003, which means that it had recovered from a previous infection, and had both IgM and IgG on 17 December 2003, which means that it had been recently reinfected. It did not show any clinical sign of myxomatosis, showing that this reinfection was asymptomatic. Rabbit A92 (adult) had only IgG on 21 February 2003 and had both IgM and IgG on 21 November 2003 and on 20 January 2004. Since IgM are detectable during about 3 weeks, it is likely that this rabbit had been reinfected twice with about 2 months between the two reinfections. Among the 9 cases of reinfection proved by the data, only one showed clinical signs of mild myxomatosis and two were considered recovered. These reinfections were therefore asymptomatic or with symptoms of attenuated myxomatosis.

**Table 2:** Changes in the serological status of rabbits caught several times during the study

**Rabbit**	**Sex**	**Date**	**Weight (g)**	**Clinical signs**	**IgM**	**IgG**	**Reinfection**
A103	F	21/03/2003	1760	None	-	-	
		13/05/2003	1700	None	-	+	
		19/03/2004	1900	Mild	+	+	Yes
A132	F	23/09/2003	1760	None	-	+	
		17/12/2003	1700	None	+	+	Yes
A135	M	24/09/2003	1200	None	-	+	
		16/12/2003	1350	None	+	+	Yes
		20/01/2004	1200	None	+	+	Possible
A136	F	24/09/2003	1400	None	-	+	
		14/05/2004	1300	Recovered	+	+	Yes
A144	M	16/10/2003	480	None	-	+	
		17/12/2003	1100	None	+	+	
		20/01/2004	1120	None	+	+	Possible
A55	M	21/08/2002	1400	None	+	+	
		12/09/2002	1400	None	+	+	Possible
A92	M	21/02/2003	1360	None	-	+	
		21/11/2003	1400	None	+	+	Yes
		20/01/2004	1430	None	+	+	Yes
SB34	F	26/06/2002	1500	None	+	+	
		17/07/2002	1500	None	+	+	Possible
SB40	M	05/07/2002	1200	None	-	+	
		27/01/2004	1480	None	+	+	Yes
SB74	F	30/09/2002	1400	None	+	+	
		21/11/2002	1300	None	+	+	Yes
SB78	M	21/01/2003	1440	Mild	+	+	
		13/01/2004	1550	Recovered	+	+	Yes
SB87	M	24/06/2003	600	None	+	+	
		22/07/2003	920	None	+	+	Possible

When rabbits carried IgM at two consecutive captures we considered that it was the same infection if the time interval between the captures was less than 1 month. It was considered as a reinfection when the time interval between the captures was more than 1.5 month. In the other cases it was considered as a possible reinfection.

### Herd immunity and circulation of the virus

**Figure 2:**
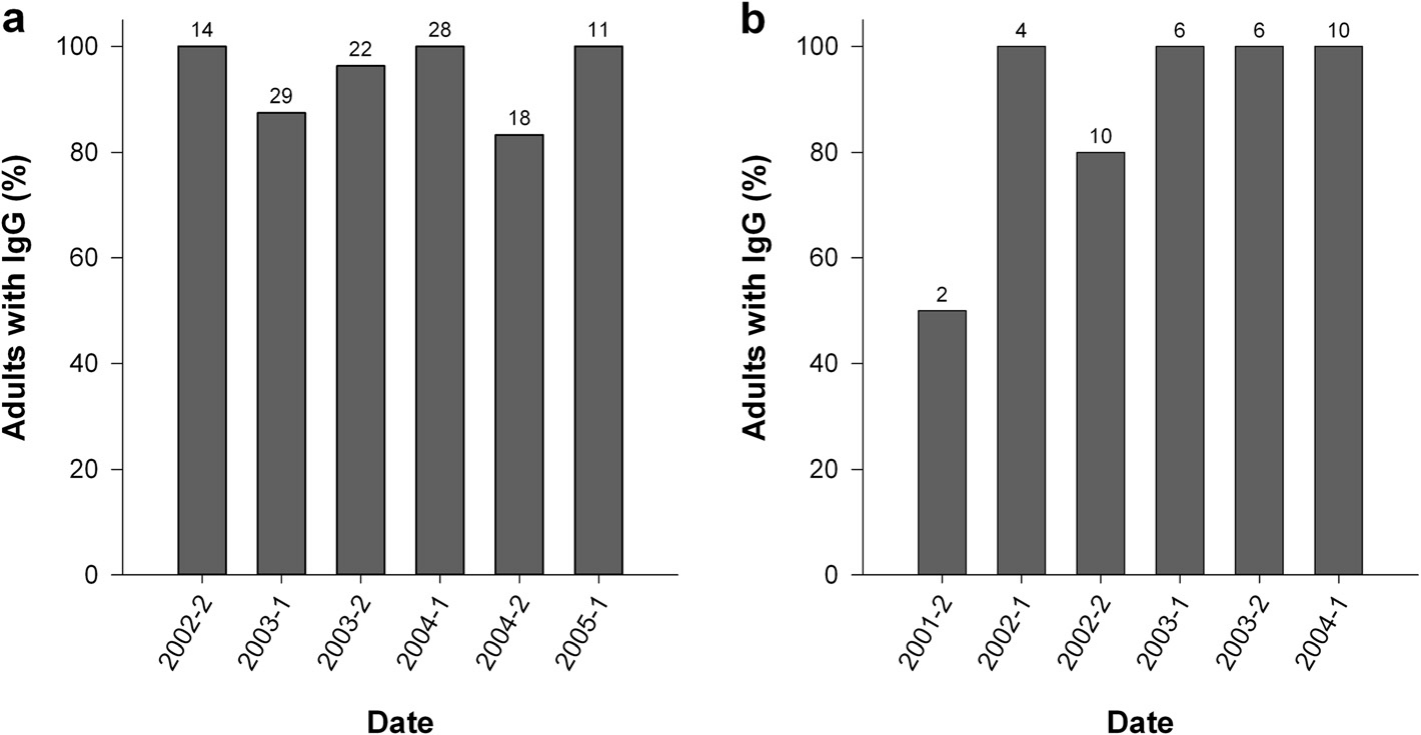
**Temporal changes in the proportion of adults with IgG.** Aubas: **a**. St Benoist: **b**. Data are pooled in six-month periods. Sample sizes are indicated above the bars. For rabbits caught several times during the same period the data from the first capture was taken into account.

**Figure 3:**
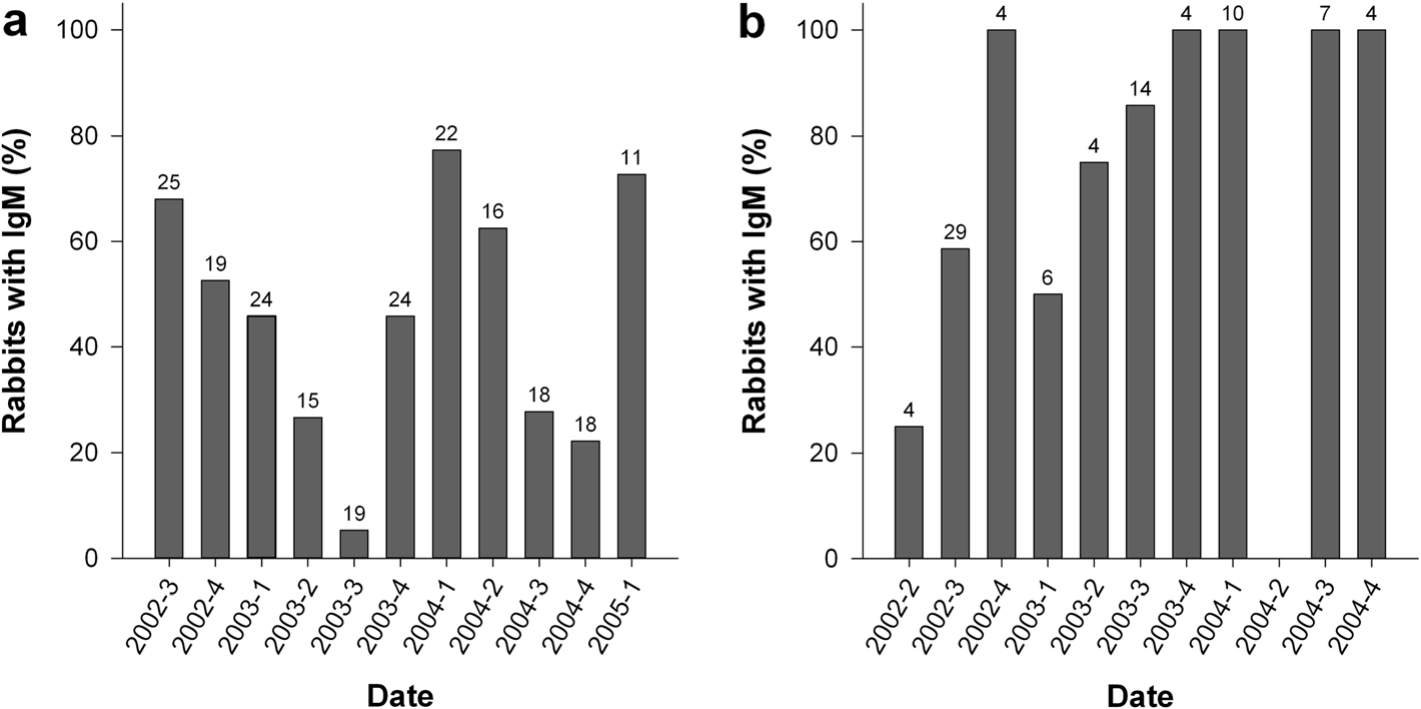
**Temporal changes in the proportion of rabbits with IgM.** Aubas: **a**. St Benoist: **b**. Data are pooled in three-month periods. Sample sizes are indicated above the bars. No rabbit was caught in 2004–2 in St Benoist.

Due to small sample sizes, data on immunity in adults were pooled in six-month periods for both areas. We observed that the immunity level in the adult population was permanently high on the two areas. Between July 2002 and June 2005, the proportion of adults with antibodies varied between 83 and 100% in Aubas. Between January 2002 and June 2004, it varied from 50 to 100% in Saint Benoist (Figure 2). We used the proportion of IgM positive rabbits, both juveniles and adults, as an indicator of the virus circulation by three-month periods (Figure 3). We recorded numerous cases of recent infection that were distributed throughout the study period since some rabbits were caught with IgM in all seasons of all years. However the proportion of rabbits with IgM changed over time, which shows that the intensity of virus circulation was not constant.

### Low prevalence of the disease

Overall, in Aubas, only 6.0% of adults (8/133) and 1.1% of juveniles (1/91) were caught with clinical signs of myxomatosis: three adults had signs of mild disease, the five others had recovered and the juvenile showed signs of mild myxomatosis. In Saint Benoist, 7.7% of adults (3/39) were caught with clinical signs: two had severe myxomatosis and one had recovered. No juvenile among 41 was caught with clinical signs in Saint Benoist.

## Discussion

Our data highlight early infections without severe disease in juveniles, which gives support to Zinkernagel’s mechanism regarding the role of maternal immunity. Indeed, despite being constrained by the difficulty of sampling in wild populations and by some uncontrolled factors, such as the viral strains circulating in the populations, they allowed us to study the relevance of our predictions on the mechanisms of establishment of immunity in juveniles and to describe the pattern of virus circulation and the impact of the disease to the individual and population levels under natural conditions [55].

One may assume that the low prevalence of the disease, i.e. the proportion of individuals showing clinical signs of myxomatosis, was due to the low virulence of MYXV strains that have circulated in both of the rabbit populations studied. Since there is no genetic characteristic of MYXV related to virulence [38], strain virulence is classically determined by in vivo experiments [26]. We did not conduct these experiments for ethical and biological reasons. First, obtaining a complete inventory of circulating MYXV strains in both populations would have required inoculating a huge number of rabbits. Second, since myxomatosis was mainly asymptomatic in most cases, it was not possible to identify rabbits to sample in order to collect the virus. However, since lowly virulent strains (grade V according to Fenner and Fantini [26]) represent less than 5% of the MYXV strains circulating in wild rabbits in Europe [26], it is unlikely that such strains have circulated alone over three years in two distant rabbit populations. Therefore, we dismiss this hypothesis and we conclude that the low prevalence of the disease is linked to rabbit immunity and to the permanent circulation of MYXV.

Rabbits less than 8 weeks old carrying IgM, which indicates a recent infection, only developed an asymptomatic or a mild disease, suggesting that they were partly protected by maternal antibodies when infected. Juveniles are known to be highly susceptible to myxomatosis and show severe disease whatever the MYXV strain involved [49,50]. These observations show that, in the rabbit/myxomatosis system, maternal antibodies do not prevent rabbits from being infected but attenuate infection and do not prevent an immune response from infected juveniles. Our data therefore support Zinkernagel’s hypothesis in accordance with similar observations made on other host/parasite systems [17-20,23-25].

Moreover, these data point out that most juveniles had maternal antibodies. The proportion of rabbits less than 400 g with IgG is high in both populations. If these IgG were only acquired, one would have expected to observe juveniles with signs of severe myxomatosis, which was not the case. This indicates that either they had not been infected, implying that these IgG are of maternal origin, or they had been infected without showing any clinical sign because they were protected by maternal antibodies when infected. One cannot exclude that some severe cases of myxomatosis remained undetected in juveniles before their emergence from the nest since wild rabbits cannot be captured at this age. However, these severe cases are probably quite rare because of the high proportion of immune adults and consequently of the high proportion of maternally protected juveniles.

In addition, we showed that infections and reinfections occur in all seasons, and that reinfections reactivate the immune system of rabbits without any severe disease. These reinfections may explain some results obtained in other studies where rabbits remained seropositive over periods up to 36 months [34]. Our results also confirm that secondary exposures to a poxvirus may induce an IgM response, as previously observed during the 2003 North American outbreak of Monkeypox for people who were vaccinated against smallpox as children [56].

These observations enable us to account for the epidemiological pattern of the disease in the populations studied. They validate the expectations of the models developed by Fouchet et al. [9,11] for analysing the relationship between virus persistence and impact of myxomatosis in wild populations under Zinkernagel’s scenario. They show that when the virus continuously circulates, individuals are steadily infected and develop mild or asymptomatic diseases that enable the reactivation of their immune system. As soon as the proportion of immune adults is high, numerous young are born with maternal antibodies. If infected before maternal immunity wanes, which is frequent with the steady circulation of the virus, they also develop an attenuated disease that leads to the development of their own specific immune response. In such endemic conditions, the disease has a low impact. In addition to the mechanisms described by Fouchet et al. [9,11], one cannot rule out that long-term effects of maternal immunity [23-25] have contributed to establish a strong herd immunity and to reduce the impact of the disease in the two populations studied.

To conclude, this work highlights the role of maternal antibodies in the establishment of strong herd immunity in wild populations. It describes the mechanism whereby, together with changes in virulence of MYXV and with the increase of genetic resistance in rabbits, the development of immunity in rabbit populations is responsible for a decrease of the impact of myxomatosis [26]. The relevance of Zinkernagel’s mechanism for the rabbit/myxomatosis system gives strength to the predictions of Fouchet et al. [9-11]: population size, population structure but also demographic parameters such as the length of the breeding season play a major role in virus persistence, which enables the maintenance of high immunity levels. This means that the variability of impact of myxomatosis among populations is not only driven by the coevolution between the virus and its host, but also by population dynamics and the large scale spatial structure of the populations.

## Competing interests

The authors declare that they have no competing interest.

## Authors’ contributions

Study development and design: SM, DP, SB, JL, JSG, DF. Field work: JA, FB, YL, AR, SM. Serological analyses: JG, BP, SB. Data analyses: SM, JL, JSG. Drafting of the paper: SM, DP, SB, JL, JSG, DF. All the authors read and approved the final manuscript.

## Supplementary Material

Additional file 1 **Full data set.** Adults: A; Juveniles: J; missing data*. Click here for file
