# Supplementary material for: Early infections by myxoma virus of young rabbits (Oryctolagus cuniculus) protected by maternal antibodies activate their immune system and enhance herd immunity in wild populations
Source: Vet Res. 2014 Mar 4;45(1):26. doi: 10.1186/1297-9716-45-26 (PMC4014812; doi:10.1186/1297-9716-45-26)
Supplement: Additional file 1 — Full data set. Adults: A; Juveniles: J; missing data*. [file 1297-9716-45-26-S1.doc]

| Rabbit | Date | Weight (g) | Age | IgG | IgM |
| --- | --- | --- | --- | --- | --- |
| A100 | 19/03/2003 | 230 | J | + | + |
| A101 | 19/03/2003 | 1550 | A | + | + |
| A102 | 20/03/2003 | 1400 | A | + | + |
| A103 | 21/03/2003 | 1760 | A | - | - |
| A103 | 13/05/2003 | 1700 | A | + | - |
| A103 | 19/03/2004 | 1900 | A | + | + |
| A104 | 21/03/2003 | 1600 | A | + | + |
| A104 | 09/04/2003 | 1600 | A | + | + |
| A107 | 10/04/2003 | 500 | J | - | - |
| A108 | 11/04/2003 | 500 | J | + | + |
| A109 | 13/05/2003 | 1300 | A | + | + |
| A11 | 20/02/2003 | 1500 | A | + | - |
| A111 | 14/10/2003 | 1600 | A | + | - |
| A112 | 14/05/2003 | 1530 | A | + | - |
| A113 | 15/05/2003 | 1750 | A | + | - |
| A114 | 15/05/2003 | 960 | J | + | - |
| A115 | 16/05/2003 | 1080 | J | + | + |
| A116 | 23/06/2003 | 200 | J | - | - |
| A117 | 24/06/2003 | 300 | J | - | - |
| A119 | 27/06/2003 | 1050 | J | - | - |
| A120 | 22/07/2003 | 1200 | J | - | - |
| A122 | 24/07/2003 | 1350 | J | + | - |
| A123 | 29/07/2003 | 1000 | J | + | - |
| A125 | 26/08/2003 | 1050 | J | + | - |
| A126 | 26/08/2003 | 1090 | J | + | - |
| A127 | 26/08/2003 | 1100 | J | + | - |
| A128 | 27/08/2003 | 1250 | A | - | - |
| A128 | 14/10/2003 | 1300 | A | + | - |
| A129 | 28/08/2003 | 850 | J | + | - |
| A130 | 29/08/2003 | 1350 | A | + | - |
| A130 | 14/10/2003 | 1450 | A | + | - |
| A131 | 01/09/2003 | 1450 | A | + | - |
| A131 | 24/09/2003 | 1450 | A | + | - |
| A132 | 23/09/2003 | 1750 | A | + | - |
| A132 | 17/12/2003 | 1700 | A | + | + |
| A133 | 23/09/2003 | 1560 | A | + | - |
| A133 | 16/10/2003 | 1300 | A | + | - |
| A134 | 23/09/2003 | 1250 | J | + | - |
| A135 | 24/09/2003 | 1200 | J | + | - |
| A135 | 16/12/2003 | 1350 | A | + | + |
| A135 | 20/01/2004 | 1200 | A | + | + |
| A136 | 24/09/2003 | 1400 | J | + | - |
| A136 | 14/05/2004 | 1300 | A | + | + |
| A137 | 25/09/2003 | 1160 | J | + | + |
| A137 | 13/05/2004 | 1450 | A | + | + |
| A137 | 20/01/2005 | 1310 | A | + | + |
| A138 | 26/09/2003 | 1200 | A | + | - |
| A139 | 14/10/2003 | 1400 | A | + | - |
| A139 | 31/07/2004 | 1600 | A | + | + |
| A140 | 14/10/2003 | 1200 | J | + | - |
| A141 | 15/10/2003 | 1320 | A | + | + |
| A142 | 16/10/2003 | 1550 | A | + | - |
| A143 | 16/10/2003 | 400 | J | + | - |
| A144 | 17/10/2003 | 480 | J | + | - |
| A144 | 17/12/2003 | 1100 | J | + | + |
| A144 | 20/01/2004 | 1120 | A | + | + |
| A145 | 17/10/2003 | 1260 | A | + | - |
| A147 | 18/11/2003 | 400 | J | + | - |
| A148 | 20/11/2003 | 400 | J | + | - |
| A149 | 21/11/2003 | 380 | J | + | - |
| A150 | 21/11/2003 | 1430 | A | + | + |
| A151 | 15/12/2003 | 1450 | A | + | + |
| A152 | 16/12/2003 | 1520 | A | + | + |
| A153 | 18/12/2003 | 1580 | A | + | + |
| A154 | 21/01/2004 | 1430 | A | + | + |
| A155 | 21/01/2004 | 1120 | A | + | + |
| A156 | 22/01/2004 | 1400 | A | + | - |
| A157 | 28/01/2004 | 1550 | A | + | + |
| A158 | 17/02/2004 | 1450 | A | + | + |
| A159 | 17/02/2004 | 1520 | A | + | + |
| A160 | 17/02/2004 | 1600 | A | + | + |
| A161 | 18/02/2004 | 1450 | A | + | + |
| A161 | 24/11/2004 | 1600 | A | + | + |
| A162 | 18/02/2004 | 1480 | A | + | + |
| A163 | 19/02/2004 | 1330 | A | + | + |
| A164 | 15/03/2004 | 520 | J | + | + |
| A166 | 16/03/2004 | 1400 | A | + | + |
| A167 | 16/03/2004 | 1800 | A | + | + |
| A167 | 11/05/2004 | 1700 | A | + | - |
| A168 | 17/03/2004 | 1560 | A | + | - |
| A169 | 17/03/2004 | 420 | J | + | - |
| A170 | 18/03/2004 | 1250 | J | + | + |
| A170 | 21/09/2004 | 1350 | J | - | - |
| A171 | 24/11/2004 | 1510 | A | + | - |
| A174 | 19/03/2004 | 1900 | A | + | - |
| A178 | 13/04/2004 | 1690 | A | + | + |
| A18 | 16/12/2003 | 1700 | A | + | + |
| A180 | 14/04/2004 | 1560 | A | + | + |
| A184 | 11/05/2004 | 520 | J | + | + |
| A185 | 11/05/2004 | 540 | J | + | - |
| A186 | 11/05/2004 | 1260 | A | + | - |
| A187 | 11/05/2004 | 1480 | A | + | + |
| A188 | 11/05/2004 | 500 | J | + | + |
| A190 | 12/05/2004 | 650 | J | + | + |
| A191 | 13/05/2004 | 1410 | A | + | + |
| A196 | 14/05/2004 | 1660 | A | + | - |
| A196 | 15/02/2005 | 1510 | A | + | + |
| A197 | 22/06/2004 | 1190 | J | - | - |
| A198 | 24/06/2004 | 1010 | J | - | - |
| A200 | 25/06/2004 | 1590 | A | + | + |
| A202 | 28/07/2004 | 1520 | A | - | - |
| A203 | 28/07/2004 | 950 | J | - | - |
| A204 | 29/07/2004 | 930 | J | - | - |
| A204 | 21/09/2004 | 1380 | J | - | - |
| A205 | 30/07/2004 | 1020 | J | - | - |
| A206 | 24/08/2004 | 800 | J | + | - |
| A207 | 24/08/2004 | 1130 | J | + | + |
| A208 | 24/08/2004 | 1200 | J | + | - |
| A209 | 25/08/2004 | 890 | J | + | + |
| A209 | 18/01/2005 | 1110 | A | + | + |
| A210 | 26/08/2004 | 1290 | J | + | - |
| A210 | 21/01/2005 | 1320 | A | + | + |
| A211 | 26/08/2004 | 1380 | J | + | - |
| A212 | 26/08/2004 | 1390 | J | + | - |
| A213 | 27/08/2004 | 1560 | A | + | - |
| A214 | 20/09/2004 | 1510 | A | + | + |
| A217 | 22/09/2004 | 1600 | A | + | - |
| A218 | 23/09/2004 | 1300 | J | - | - |
| A220 | 26/10/2004 | 1250 | J | - | - |
| A221 | 27/10/2004 | 1200 | J | - | - |
| A222 | 28/10/2004 | 1300 | J | - | - |
| A223 | 29/10/2004 | 1440 | A | - | - |
| A224 | 23/11/2004 | 840 | J | + | + |
| A225 | 24/11/2004 | 1400 | A | + | + |
| A226 | 24/11/2004 | 1410 | A | + | - |
| A226 | 19/01/2005 | 1490 | A | + | - |
| A228 | 24/11/2004 | 1290 | J | + | - |
| A229 | 25/11/2004 | 1320 | A | + | - |
| A230 | 25/11/2004 | 1220 | J | - | - |
| A230 | 14/12/2004 | 1310 | A | - | - |
| A231 | 13/12/2004 | 1300 | A | + | - |
| A232 | 14/12/2004 | 1310 | A | + | - |
| A233 | 14/12/2004 | 1360 | A | + | - |
| A234 | 15/12/2004 | 1520 | A | + | - |
| A235 | 15/12/2004 | 1330 | A | + | - |
| A236 | 16/12/2004 | 1480 | J | + | + |
| A237 | 19/01/2005 | 1360 | A | + | - |
| A238 | 19/01/2005 | 1540 | A | + | - |
| A239 | 15/02/2005 | 1590 | A | + | + |
| A240 | 18/02/2005 | 1600 | A | + | + |
| A241 | 18/02/2005 | 1610 | A | + | + |
| A31 | 22/01/2003 | 1500 | A | + | - |
| A36 | 18/06/2002 | 930 | J | + | + |
| A38 | 18/06/2002 | 320 | J | - | - |
| A40 | 19/06/2002 | 400 | J | - | - |
| A41 | 19/06/2002 | 360 | J | - | - |
| A44 | 21/06/2002 | 800 | J | + | - |
| A45 | 22/07/2002 | 450 | J | + | + |
| A46 | 23/07/2002 | 1650 | A | + | + |
| A47 | 23/07/2002 | 400 | J | + | + |
| A48 | 23/07/2002 | 400 | J | + | + |
| A50 | 25/07/2002 | 850 | J | + | + |
| A51 | 26/07/2002 | 450 | J | - | - |
| A52 | 26/07/2002 | 500 | J | + | + |
| A53 | 20/08/2002 | 820 | J | + | + |
| A54 | 21/08/2002 | 1050 | J | + | + |
| A55 | 21/08/2002 | 1400 | A | + | + |
| A55 | 12/09/2002 | 1400 | A | + | - |
| A56 | 21/08/2002 | 230 | J | - | - |
| A57 | 22/08/2002 | 720 | J | + | - |
| A58 | 24/09/2002 | 350 | J | + | - |
| A59 | 24/09/2002 | 1130 | A | + | + |
| A60 | 24/09/2002 | 1150 | J | + | + |
| A63 | 25/09/2002 | 1250 | J | - | - |
| A64 | 26/09/2002 | 1100 | J | + | + |
| A65 | 26/09/2002 | 1250 | A | + | + |
| A66 | 26/09/2002 | 1200 | J | + | - |
| A67 | 26/09/2002 | 850 | J | + | + |
| A68 | 26/09/2002 | 1300 | A | + | + |
| A68 | 20/11/2002 | 1350 | A | + | + |
| A69 | 27/09/2002 | 1100 | A | + | + |
| A70 | 22/10/2002 | 730 | J | + | - |
| A70 | 20/11/2002 | 800 | J | + | - |
| A71 | 22/10/2002 | 800 | J | + | + |
| A71 | 20/03/2003 | 1400 | A | + | - |
| A72 | 24/10/2002 | 1350 | A | + | - |
| A73 | 24/10/2002 | 700 | J | + | - |
| A74 | 21/11/2002 | 1550 | A | + | - |
| A74 | 26/06/2003 | 1400 | A | + | - |
| A75 | 21/11/2002 | 1250 | J | + | - |
| A76 | 21/11/2002 | 820 | J | + | - |
| A76 | 22/01/2003 | 1250 | A | + | - |
| A76 | 25/06/2003 | 1400 | A | + | - |
| A76 | 26/09/2003 | 1200 | A | + | - |
| A77 | 22/11/2002 | 1650 | A | + | + |
| A77 | 22/01/2003 | 1550 | A | - | - |
| A77 | 21/01/2004 | 1450 | A | + | - |
| A77 | 27/08/2004 | 1500 | A | + | + |
| A78 | 22/11/2002 | 1400 | A | + | + |
| A79 | 04/12/2002 | 1220 | A | + | - |
| A8 | 25/06/2003 | 1450 | A | + | - |
| A80 | 05/12/2002 | 1350 | A | + | - |
| A81 | 05/12/2002 | 1450 | A | + | + |
| A82 | 05/12/2002 | 1350 | A | + | + |
| A82 | 19/01/2005 | 1380 | A | + | + |
| A83 | 06/12/2002 | 1350 | J | + | + |
| A84 | 06/12/2002 | 1000 | J | + | + |
| A85 | 22/01/2003 | 1500 | A | + | - |
| A86 | 23/01/2003 | 1700 | A | + | - |
| A86 | 01/09/2003 | 1700 | A | + | - |
| A87 | 23/01/2003 | 1300 | A | + | + |
| A88 | 23/01/2003 | 1100 | A | + | + |
| A88 | 17/12/2003 | 1650 | A | + | + |
| A89 | 24/01/2003 | 1400 | A | + | + |
| A90 | 24/01/2003 | 1650 | A | + | - |
| A91 | 24/01/2003 | 1400 | A | + | - |
| A92 | 21/02/2003 | 1360 | A | + | - |
| A92 | 21/11/2003 | 1400 | A | + | + |
| A92 | 20/01/2004 | 1430 | A | + | + |
| A93 | 21/02/2003 | 1400 | A | + | + |
| A94 | 18/03/2003 | 1650 | A | + | - |
| A95 | 18/03/2003 | 1350 | A | + | + |
| A96 | 18/03/2003 | 1650 | A | - | - |
| A97 | 19/03/2003 | 1700 | A | - | - |
| A98 | 19/03/2003 | 1350 | A | + | + |
| A99 | 19/03/2003 | 1250 | A | + | + |
| A99 | 17/10/2003 | 1340 | A | + | - |
| AM13 | 12/09/2002 | 1100 | J | + | + |
| AM14 | 12/09/2002 | 1350 | J | - | - |
| AM15 | 12/09/2002 | 1200 | J | - | - |
| AM19 | 31/10/2002 | 1050 | J | + | + |
| AM20 | 31/12/2002 | 1100 | J | + | - |
| AM21 | 31/12/2002 | 1050 | J | + | + |
| SB1 | 05/12/2001 | 1800 | A | - | - |
| SB1 | 18/07/2002 | 1600 | A | + | + |
| SB10 | 29/07/2003 | * | A | + | + |
| SB100 | 09/09/2003 | 1500 | A | + | - |
| SB101 | 09/09/2003 | 900 | J | + | + |
| SB103 | 10/09/2003 | * | * | + | + |
| SB105 | 11/12/2003 | 1400 | A | + | + |
| SB106 | 12/12/2003 | 1300 | A | + | + |
| SB108 | 13/01/2004 | 1500 | A | + | + |
| SB109 | 28/01/2004 | 1200 | A | + | + |
| SB110 | 05/02/2004 | 1050 | A | + | + |
| SB111 | 05/02/2004 | 1300 | A | + | + |
| SB112 | 05/02/2004 | 1620 | A | + | + |
| SB113 | 10/02/2004 | * | A | + | + |
| SB114 | 10/02/2004 | 1500 | A | + | + |
| SB114 | 02/03/2004 | * | A | + | + |
| SB115 | 19/02/2004 | 1600 | A | + | + |
| SB117 | 21/07/2004 | 900 | J | + | + |
| SB118 | 21/07/2004 | 1150 | J | + | + |
| SB119 | 23/07/2004 | 1170 | * | + | + |
| SB120 | 28/07/2004 | 1150 | * | + | + |
| SB121 | 28/07/2004 | 280 | J | + | + |
| SB122 | 05/08/2004 | 1500 | A | + | + |
| SB123 | 12/08/2004 | 1150 | J | + | + |
| SB125 | 03/11/2004 | 1200 | * | + | + |
| SB126 | 03/11/2004 | 1200 | * | + | + |
| SB127 | 04/11/2004 | 1350 | * | + | + |
| SB128 | 05/11/2004 | 1350 | * | + | + |
| SB15 | 21/02/2002 | 1420 | A | + | - |
| SB2 | 05/12/2001 | 1250 | A | + | - |
| SB28 | 28/05/2002 | 900 | J | + | - |
| SB31 | 19/06/2002 | 1360 | A | + | - |
| SB33 | 25/06/2002 | 1600 | A | + | - |
| SB34 | 26/06/2002 | 1500 | A | + | + |
| SB34 | 17/07/2002 | 1500 | A | + | + |
| SB38 | 04/07/2002 | 1530 | A | + | - |
| SB39 | 05/07/2002 | 1330 | A | + | - |
| SB40 | 05/07/2002 | 1200 | A | + | - |
| SB40 | 27/01/2004 | 1480 | A | + | + |
| SB41 | 09/07/2002 | 750 | J | + | - |
| SB44 | 17/07/2002 | 590 | J | + | + |
| SB45 | 23/07/2002 | 1100 | J | + | + |
| SB46 | 23/07/2002 | 920 | J | + | + |
| SB47 | 23/07/2002 | 890 | J | + | + |
| SB48 | 23/07/2002 | 510 | J | + | + |
| SB52 | 25/07/2002 | 1200 | J | + | + |
| SB53 | 26/07/2002 | 1200 | J | + | - |
| SB54 | 26/07/2002 | 810 | J | + | - |
| SB55 | 26/07/2002 | 940 | J | + | + |
| SB56 | 08/08/2002 | * | J | + | + |
| SB57 | 06/08/2002 | 980 | J | + | - |
| SB59 | 21/08/2002 | 990 | J | + | - |
| SB60 | 14/08/2002 | 1100 | J | + | + |
| SB63 | 13/08/2002 | 1200 | J | + | + |
| SB64 | 14/08/2002 | 700 | J | + | - |
| SB65 | 15/08/2002 | 490 | J | + | + |
| SB66 | 21/08/2002 | 1210 | A | + | - |
| SB68 | 20/09/2002 | 400 | J | + | + |
| SB71 | 27/09/2002 | 770 | J | - | - |
| SB72 | 30/09/2002 | 1400 | A | - | - |
| SB73 | 30/09/2002 | 1720 | A | - | + |
| SB74 | 30/09/2002 | 1400 | J | + | + |
| SB74 | 21/11/2002 | 1300 | A | + | + |
| SB75 | 15/10/2002 | 1120 | J | + | + |
| SB76 | 12/11/2002 | 1380 | J | + | + |
| SB78 | 21/01/2003 | 1440 | A | + | + |
| SB78 | 13/01/2004 | 1550 | A | + | + |
| SB79 | 21/01/2003 | 1440 | A | + | + |
| SB80 | 21/01/2003 | 1300 | A | + | - |
| SB81 | 22/01/2003 | 1400 | A | + | + |
| SB82 | 04/02/2003 | 1300 | A | + | - |
| SB83 | 15/10/2003 | 1200 | A | + | + |
| SB85 | 26/02/2003 | * | A | + | - |
| SB86 | 24/06/2003 | 600 | J | + | + |
| SB87 | 24/06/2003 | 600 | J | + | + |
| SB87 | 22/07/2003 | 920 | J | + | + |
| SB88 | 25/06/2003 | 410 | J | + | + |
| SB89 | 25/06/2003 | 260 | J | + | - |
| SB90 | 08/07/2003 | 670 | J | + | + |
| SB91 | 11/07/2003 | 550 | J | + | + |
| SB92 | 23/07/2003 | 880 | J | + | + |
| SB94 | 25/07/2003 | 650 | J | + | + |
| SB95 | 29/07/2003 | 230 | J | - | + |
| SB97 | 06/08/2003 | 1200 | J | + | + |
| SB98 | 06/08/2003 | 400 | J | + | - |
| SB99 | 01/09/2003 | 1700 | A | + | + |
| SBM1 | 03/07/2002 | 1600 | A | + | + |
| SBM10 | 05/10/2003 | 650 | J | + | + |
| SBM11 | 29/09/2003 | 1200 | * | + | + |
| SBM8 | 30/10/2002 | * | * | + | + |
